# Supplementary material for: Physicians’ attitude towards webinars and online education amid COVID-19 pandemic: When less is more
Source: PLoS One. 2021 Apr 16;16(4):e0250241. doi: 10.1371/journal.pone.0250241 (PMC8051773; doi:10.1371/journal.pone.0250241)
Supplement: S1 Appendix — (DOCX) [file pone.0250241.s001.docx]

**S1 Appendix. Survey Questions**

1. **What is your age?**
2. **What is your gender?**Male
   Female
3. **What is your country of practice?**
4. **What is your medical/ surgical specialty?**
5. **How many years of practice do you have in your specialty?**
6. **How do you describe your role amid COVID-19 pandemic?**Frontline healthcare worker
   I continued to practice my usual work during the pandemic
   I didn't work during the pandemic
   I partially worked during the pandemic
   I practiced through "telemedicine" services
7. **Have you attended webinars or online meetings during the past 6 months?**No
   Yes
8. **If yes, how many webinars or online meetings, in average, have you attended per month during the past 6 months?**
9. **How many webinars or online meetings, in average, have you attended per month last year?**
10. **In comparison to the last year, have you attended more meetings and conferences during the past 6 months?**No
    Maybe
    Yes
11. **What is your role in webinars and online meetings?**Equally speaker and attendee
    Mostly attendee
    Mostly speaker
12. **In average, what is the percentage of webinars or online meetings you attend in comparison to the number you are invited to?**Less than 25%
    25 – 50%
    50 – 75%
    More than 75 %
13. **Since the beginning of COVID-19 pandemic, what is your general impression on shifting scientific meetings to webinars and online meetings?**Strongly dissatisfied
    Dissatisfied
    Neutral
    Satisfied
    Strongly satisfied
14. **What is your impression on shifting international conferences to webinars and online meetings?**Strongly dissatisfied
    Dissatisfied
    Neutral
    Satisfied
    Strongly satisfied
15. **What is your impression on shifting teaching courses to webinars and online meetings?**Strongly dissatisfied
    Dissatisfied
    Neutral
    Satisfied
    Strongly satisfied
16. **What is your impression on shifting pharmaceutically-sponsored meetings to webinars and online meetings?**Strongly dissatisfied
    Dissatisfied
    Neutral
    Satisfied
    Strongly satisfied
17. **In general, what is your impression on the scientific content of the International conferences as webinars and online meetings?**Strongly dissatisfied
    Dissatisfied
    Neutral
    Satisfied
    Strongly satisfied
18. **In general, what is your impression on the scientific content of the pharmaceutically-sponsored meetings as webinars and online meetings?**Strongly dissatisfied
    Dissatisfied
    Neutral
    Satisfied
    Strongly satisfied
19. **What type of online meetings do you usually prefer to attend?**International conferences
    Local/ regional conferences,
    Teaching/ training courses
    Commercial/ pharmaceutically sponsored webinars
20. **What factors determine which webinar or online meeting you attend?**Scientific content of the meeting
    Speaker's name and experience
    International annual conferences
    CME certification
    Personal relationship with the inviting party
    Other factors
21. **What factors determine which webinar or online meeting you reject or refuse to attend?**Timing of the meeting
    Topics of the meeting
    Speaker's in the meeting
    Lack of CME certification,
    Lack of personal relationship with the inviting party
    Other factors
22. **Do you agree that webinars and online meetings can replace in-person meetings after the pandemic?**Strongly disagree
    Disagree
    Neutral
    Agree
    Strongly agree
23. **Have you felt overwhelmed with the number and frequency of webinars and online meetings during the pandemic?**Strongly disagree
    Disagree
    Neutral
    Agree
    Strongly agree
24. **Do you agree that webinars and online meetings need further regulations?**Strongly disagree
    Disagree
    Neutral
    Agree
    Strongly agree
25. **If you strongly agree, or agree, please specify how do you think these meetings can be regulated in the future?**
