# Supplementary material for: Physicians’ attitude towards webinars and online education amid COVID-19 pandemic: When less is more
Source: PLoS One. 2021 Apr 16;16(4):e0250241. doi: 10.1371/journal.pone.0250241 (PMC8051773; doi:10.1371/journal.pone.0250241)
Supplement: Attachment — Submitted filename: RESPONSE to editor and reviewers PLOS R2.docx [file pone.0250241.s003.docx]

**Response to editor and reviewers’ comments**

**Comment to Editor**

**Dear Editor**

**Thank you for evaluating our manuscript. We have addressed the comments of the reviewers at this point. There have been some comments to modify the title. Despite having a majority of responses from Arab and MENA countries, we still had 10% of the responses from Western countries. Another point that was raised is to change the word “attitude” in the title. We think the current title represents the essence of the work, and we would like to leave the decision on this matter in your hands.**

**Review Comments to the Author**

**Reviewer #1: (No Response)**

**Reviewer #4:**

Dear Respected Authors,
Thanks for your efforts trying to improve your research manuscript according to the reviewers' comments and suggestions.
Although you have mentioned you agree that a valid scale needed to be able to measure the "attitude" of physicians, you did not made any changes or improvements in neither title nor text of your manuscript.

**We would like to thank the respected reviewer for the constructive remarks. We agree with the reviewer that our survey was not validated, and this was mentioned in the Limitations section. We used 5-point Likert scale of agreeableness which has been used in several studies to measure attitude towards different variables. We updated the limitations section as regard to the reliability in measuring attitude.**

Pilot study is still unclear in terms of method and findings in the manuscript.

**Each author sent the questionnaire to a random of 10 physicians of his contacts, and 25 responded, which was considered a convenient sample to this pilot. The Methods section was updated (Line 129-134), highlighted in green.**

Best regards

**Reviewer #5:**

Thank you for your resubmission of your publication. I have several comments, mostly relating to my initial evaluation. One new comment is in regards to your statistical analysis in Table 3 and 4. I am not sure what your comparisons are for each question. You used a Goodness of Fit, but I'm not sure what your comparison values were. Any statistical analysis on these responses doesn't seem necessary.

**We would like to thank the reviewer for the constructive remarks. These tables were added upon your suggestions to add (p value) in describing the responses. However, we can remove them and keep the p values only, if this is recommended and acceptable by the journal editor.**

Regarding your survey population, I believe the response group should be noted in the title or abstract.

**We added this point to the abstract (highlighted in green) as suggested. We focused on this point in the Discussion as requested. Also, this was mentioned in the Limitations part.**

For your recommendations at the end of the paper, it would be helpful if you referenced the other publications that recommendations not directly related to your study are from. You mention that some of the recommendations are from feedback in your survey; could you add the specific text they provided? (ie. Recommendation 2 does not cite the publication for preference for Tuesday; Recommendation 3 mentions previous work on seminar length but does not cite it; 5-7 seem to be more opinions)

**We updated this part in the manuscript and assigned the references used for these recommendations.**
